# Supplementary material for: Interplay between spherical confinement and particle shape on the self-assembly of rounded cubes
Source: Nat Commun. 2018 Jun 8;9:2228. doi: 10.1038/s41467-018-04644-4 (PMC5994693; doi:10.1038/s41467-018-04644-4)
Supplement: Supplementary file 1 — Supplementary Information [file 41467_2018_4644_MOESM1_ESM.pdf]

Interplay between spherical confinement and particle shape on the self-assembly of rounded cubes

Wang *et al.*

Supplementary Methods

Section 1 Chemicals

Chemicals used were dextran from Leuconostoc mesenteroides (Sigma Aldrich, mol. wt. 1.500.000-2.800.000), cyclohexane (Sigma Aldrich, 99.8%), n-hexane (Sigma Aldrich, ≥99.5%), 1-octadecene (1-ODE, Sigma Aldrich, 90%) Sodium dodecyl sulfate (SDS, Sigma Aldrich, ≥99.0%), oleic acid (OA, Sigma Aldrich, ≥99.0%), sodium oleate (NaOL, TCI, >95%), isopropanol (Sigma Aldrich, ≥99.5%), methanol (Sigma Aldrich, ≥99.9%), ethanol (Baker Analyzed, ≥99.9%, absolute), iron chloride hexahydrate ($FeCl_3{\cdot}6H_2O$, Sigma Aldrich, ≥99.9%), lead (II) acetate trihydrate (Sigma Aldrich, 99.999%), $CoCl_2$ (Alfa Aesar, anhydrous), sodium hydroxide (NaOH, Sigma Aldrich, ≥98%, anhydrous), diphenylphosphine (Sigma Aldrich, >90%), trioctylphosphine (TOP, Sigma Aldrich, 97%), selenium shot (Se, Alfa Aesar, 99.999%). For de-ionized water (DI H_2O) a Millipore Direct-Q UV3 reverse osmosis filter apparatus was used (18 MΩ at 25 °C).

Section 2 Nanocrystal (NC) syntheses *and* supraparticle (SP) self-assembly (SA)

All NC syntheses were carried out under nitrogen atmosphere using standard Schlenk line techniques.

Section 2.1 Sharp nanocubes

The 22.7 nm (24.1 nm total side length due to interdigitating ligands, and a total polydispersity of 8.7%; see Supplementary Methods Section 2.3) sharp Fe_3O_4 nanocubes (Supplementary Fig. 1) were prepared by thermal decomposition of iron (III) oleate by modifying a previous literature method[1]. NaOL (36.5 g, 120 mmol) and $FeCl_3{\cdot}6H_2O$ (10.8 g, 40 mmol) were dissolved in a mixture of n-hexane (140 mL), ethanol (80 mL) and DI H_2O (60 mL). The mixture was refluxed for 4 hours at 70 °C. The organic phase was separated and washed with DI H_2O, and finally removed by a rotary evaporator at 30 °C. The obtained iron (III) oleate (0.9 g), and OA (0.14 g) were dissolved in 1-ODE (5 g) and then heated to 316 °C. After refluxing for 45 minutes, the solution was cooled down to room temperature (RT), and the obtained Fe_3O_4 nanocubes were washed with the solvent/anti-solvent pair n-hexane/isopropanol two times. Finally, the Fe_3O_4 nanocubes were weighted and redispersed in cyclohexane to a desired weight concentration.

Section 2.2 Rounded nanocubes

The 9.0 nm (10.4 nm total side length due to interdigitating ligands, and a total polydispersity of 4.1%; see Supplementary Methods Section 2.3) rounded $Fe_xO/CoFe_2O_4$ nanocubes (Supplementary Fig. 2) were synthesized according to the method described by Bodnarchuk *et al.*[2] with minor modifications. Briefly, 4.8 g of precursor stock solution, 0.38 g of OA, 0.69 g of NaOL and 6.13 g of 1-ODE were mixed and degassed at 110 °C for 60 minutes. Next, the mixture was heated to reflux under dry nitrogen. After 25 minutes, the mixture was cooled to RT, followed by

washing with the solvent/anti-solvent pair n-hexane/isopropanol two times. Finally, the $Fe_xO/CoFe_2O_4$ nanocubes were weighted and redispersed in cyclohexane to a desired weight concentration.

The 10.7 nm (12.1 nm total side length due to interdigitating ligands, and a total polydispersity of 6%) rounded PbSe nanocubes (Supplementary Fig. 3) were synthesized according to the method described by Steckel *et al.*[3]. Specifically, 0.895 g of PbO, 3.0 mL of OA, and 20 mL of 1-ODE were mixed and degassed at 105 °C for 1.5 hours. Next, the solution was heated to 210 °C in a nitrogen atmosphere. 71 µL diphenylphosphine was mixed with 8.0 mL of TOP-Se solution (1.0 M). The mixture was swiftly injected into the reaction flask. The NCs were allowed to grow at 210 °C for 30 minutes and then quenched by an ice bath. The PbSe nanocubes were purified with isopropanol three times, dispersed in cyclohexane to a desired weight concentration and stored in a glove box.

Section 2.3 Determining the total side length and shape parameter of the nanocubes

The nanocubes have a solid crystalline core with side length D_{core} that was characterized in detail by using TEM (Figs. 1b and 1c in the main text, and Supplementary Methods Sections 2.1 and 2.2). The sharp Fe_3O_4 nanocubes have an average side length of $D_{\text{core}}^{\text{sharp}} = 22.7$ nm and the rounded $Fe_xO/CoFe_2O_4$ nanocubes have an average side length of $D_{\text{core}}^{\text{rounded}} = 9.0$ nm. The ligands are not visible in the EM and will add a slightly longer ranged repulsion to the nanocubes. To quantify the effect of the ligands on the particle size we compared the structure observed in the experiments of the rounded $Fe_xO/CoFe_2O_4$ cubes with the structure obtained from the computer simulations. We defined a total side length $D = D_{\text{core}} + L$, where L is the thickness of a single interdigitated ligand layer. To obtain the total length D we compared the structures of the rounded $Fe_xO/CoFe_2O_4$ nanocubes obtained in the experiments with the structures obtained in the computer simulations. In Supplementary Fig. 13b we showed the radial distribution function as a function of the particle separation r, we aligned the experimental radial distribution with the one obtained from the computer simulations. The total side length we obtained is $D^{\text{rounded}} = 10.4 \pm 0.1$ nm. The core size of these particles as measured from TEM is $D_{\text{core}}^{\text{rounded}} = 9.0 \pm 0.1$ nm. This results in a ligand size of $L = 1.4 \pm 0.1$ nm, which is slightly shorter than the OA molecule length (∼1.8 nm)[4], indicating the ligands interdigitate between the nanocubes. As the ligands on the sharp Fe_3O_4 nanocubes had a similar size as the ligands on the rounded $Fe_xO/CoFe_2O_4$ nanocubes, the total side length of the sharp Fe_3O_4 nanocubes is $D^{\text{sharp}} = 24.1 \pm 0.1$ nm.

To obtain the particle core shape parameter α_{core} the length of the two body diagonals and the height and width of the nanocubes were determined from TEM images of a dilute layer of cubes (Figs. 1d and 1e in the main text). For the sharp Fe_3O_4 nanocubes, we found that the rounded cubic shape describes the particle shape well as shown in (Fig. 1d in the main text) (except for small variations in the aspect ratio), and we found $\alpha_{\text{core}} = 0.73 \pm 0.04$. For the rounded $Fe_xO/CoFe_2O_4$ nanocubes we obtained $\alpha_{\text{core}} = 0.35 \pm 0.04$ (Fig. 1e in the main text). To include the effect of the ligands we assumed that the ligands form a layer of constant thickness $L = 1.4$ nm around the whole particle. For the sharp Fe_3O_4 nanocubes, we found $\alpha = 0.68 \pm 0.04$. For the rounded $Fe_xO/CoFe_2O_4$ nanocubes we obtained $\alpha = 0.30 \pm 0.04$. However, in the experiment of the sharp Fe_3O_4 nanocubes we observe a simple cubic (SC) core surrounded by several shells, which we also see on the simulations but only for $\alpha > 0.75$ (see Fig. 2

in the main text). We can only speculate at the cause of this small mismatch in the particle shape. One of the possibilities is that the polydispersity or the variations in aspect ratio only present in the experiments causes the transition to shift towards lower α. Another possibility is that the surfactant coating is more complex and not of constant thickness, if for example more surfactants are anchored at the edges or corners than at the flat faces they could make the cube effectively sharper. Further experiments and simulations in which for example the surfactant is modified or polydispersity is incorporated into the simulations can shed light on this small but interesting mismatch between experiments and simulations. The high sensitivity of this system to small variations in particle shape could be used as a tool to understand more about the surfactant mediated interactions between NPs not possible to probe otherwise.

Section 2.4 Experimental SA of sharp nanocubes in spherical confinement

For a typical sharp nanocube SA in confinement experiment, 8.0 mg of sharp Fe_3O_4 nanocubes were dispersed in 1.0 mL of cyclohexane and added to a mixture of 400 mg of dextran and 23.6 mg of SDS in 10 mL of DI H_2O. The resulting emulsion was agitated by shear with a shear rate of $1.04 \times 10^5\ s^{-1}$, using a Couette rotor-stator device (gap spacing 0.100 mm) following the procedure and home-built equipment described by Mason and Bibette[5]. The emulsion was then evaporated at RT using a VWR VV3 vortex mixer for 48 hours. The resulting SPs suspension was purified by centrifugation with a speed of 2,500 rpm for 15 minutes using an Eppendorf 5415C centrifuge, followed by redispersing in DI H_2O. The above-mentioned procedure was repeated twice. Freshly prepared emulsion droplets containing sharp Fe_3O_4 nanocubes are shown in Supplementary Fig. 4. The core and the surface of the self-assembled SP are shown in Supplementary Fig. 5.

Section 2.5 Experimental SA of rounded nanocubes in spherical confinement

For a typical rounded nanocube SA in confinement experiment, 6.5 mg of rounded $Fe_xO/CoFe_2O_4$ (or PbSe) nanocubes were dispersed in 1.0 mL of cyclohexane and added to a mixture of 400 mg of dextran and 70 mg of SDS in 10 mL of DI H_2O. The resulting emulsion was agitated by shear with a shear rate of $1.56 \times 10^5\ s^{-1}$, using a Couette rotor-stator device (gap spacing 0.100 mm) following the procedure and equipment described by Mason and Bibette[5]. The emulsion was then evaporated at RT using a VWR VV3 vortex mixer for 48 hours. The resulting SPs suspension was purified by centrifugation with a speed of 2,500 rpm for 15 minutes using an Eppendorf 5415C centrifuge, followed by redispersing in DI H_2O. The above-mentioned procedure was repeated twice. Images of typical SPs consisting of rounded $Fe_xO/CoFe_2O_4$ nanocubes and PbSe nanocubes are shown in Supplementary Fig. 6 and Supplementary Fig. 8, respectively. The 3D representation of the EM tomographic reconstruction of a SP consisting of rounded $Fe_xO/CoFe_2O_4$ nanocubes is shown in Supplementary Fig. 7.

Section 3 Monte Carlo (MC) simulations

MC simulations of sharp cubes and rounded cubes were performed in the NVT and NPT ensembles (Fig. 4 in the main text and Supplementary Fig. 9). The particles were modeled as hard particles, the interaction potential V is thus given by

$$V = \begin{cases} 0 & \text{if no overlap,} \\ \infty & \text{if overlap.} \end{cases}$$

The overlap algorithm (`https://github.com/Grieverheart/ntcd`) that was used is a fast implementation of the Gilbert-Johnson-Keerthi (GJK) algorithm[6]. The particles were confined by a hard spherical shell of diameter R. The configurations were initialized with an equilibrated liquid at low volume fraction. To obtain equilibrated configurations at higher volume fractions the diameter of the confining spherical shell was reduced at a fixed rate (NVT) or the pressure was increased slowly (NPT). Compressions were performed sufficiently slow to ensure that the simulations had time to crystallize and equilibrate fully. We ensured that simulations 2 times slower and 2 times longer result in configurations

indistinguishable from the ones shown here. We observed no significant difference between the simulations in the NPT ensemble and simulations in the NVT ensemble with decreasing volume.

For sharp cubes the SPs were found to contain a SC core surrounded by shells of particles aligned with the confining interface. In Supplementary Fig. 10 we plot the fraction of particles in such shells as a function of the total number of particles. Each point is the average of 10 simulations. As a threshold for aligned with the hard interface responsible for the confinement we used an angle of 0.07 radians (or 4 degrees) between a face of the cube and the normal of the closest point on the confining sphere. We can convert the fraction f to a length scale to obtain the thickness of the layer effected by the confining surface $p = R\left(1-(1-f)^{\frac{1}{3}}\right)$, where R is the radius of the SP. This length scale p seems to grow almost linearly with the size of the SP. This counterintuitive result is probably caused by the decrease in the curvature of the interface as the SP size increases. This results in a decrease in the free energy cost of bending crystalline layers parallel to the interface.

The superstructures loose their order when the compression rate in the simulations is much higher (∼10,000 times) than the rates used to obtain equilibrated configurations. Supplementary Fig. 11 shows two typical configurations that were obtained after a fast compression.

Section 4 Connecting particle color with its orientation

It is not trivial to color the particles depending on their orientation in such a way that the same orientation results in the same color, different orientations result in different colors and small changes in orientation result in small changes in color. The orientation of a cube is described by three Euler angles (or a quaternion or a rotation matrix), but different Euler angles will result in an identical orientated cubes, which should be colored identically. We have not found a perfect solution that matches all requirements (it might not exist with only three degrees of freedom available). To obtain a visually interpretable effect we color the particles by calculating the angle between the particle and three reference particles with different orientations. We then use these three angles to obtain the red, green and blue values of the color of the particle:

$$C_i^{\text{red}} = 1 - \alpha_{i,\text{ref}_1} * 0.8, \tag{1}$$

$$C_i^{\text{green}} = 1 - \alpha_{i,\text{ref}_2} * 0.8, \tag{2}$$

$$C_i^{\text{blue}} = 1 - \alpha_{i,\text{ref}_3} * 0.8. \tag{3}$$

As reference orientations we used particles rotated $\pi/4$ around the x, y and z axis. This way we get smooth color changes, and similar orientations get a similar color, however, there are several orientations that are quite different that can get similar colors. The authors are not aware of a coloring method that does not have this problem.

Supplementary Figures

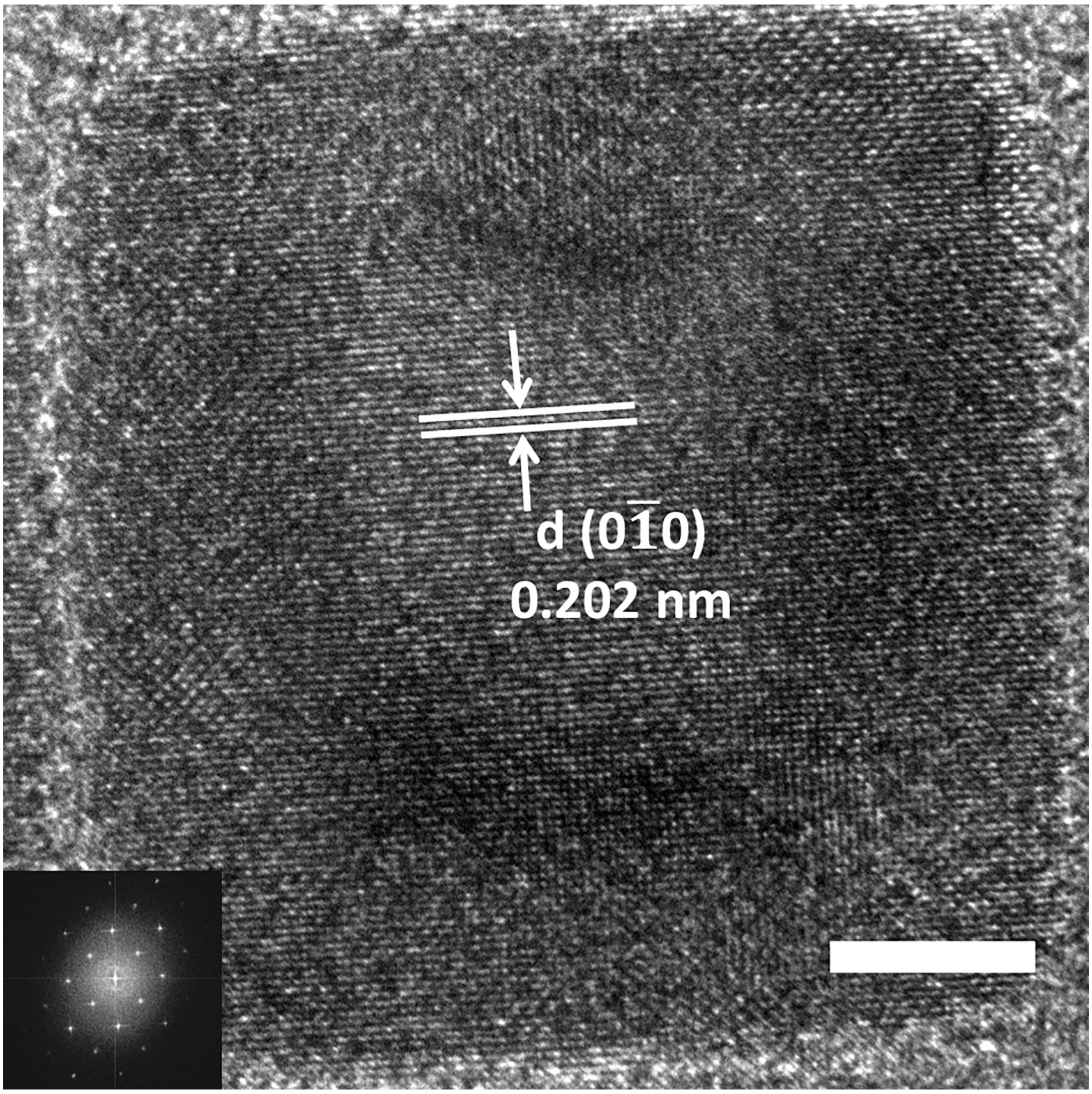

Supplementary Figure 1: HRTEM image of a sharp Fe_3O_4 nanocube. Inset, FFT of entire HRTEM image. Scale bar, 5 nm.

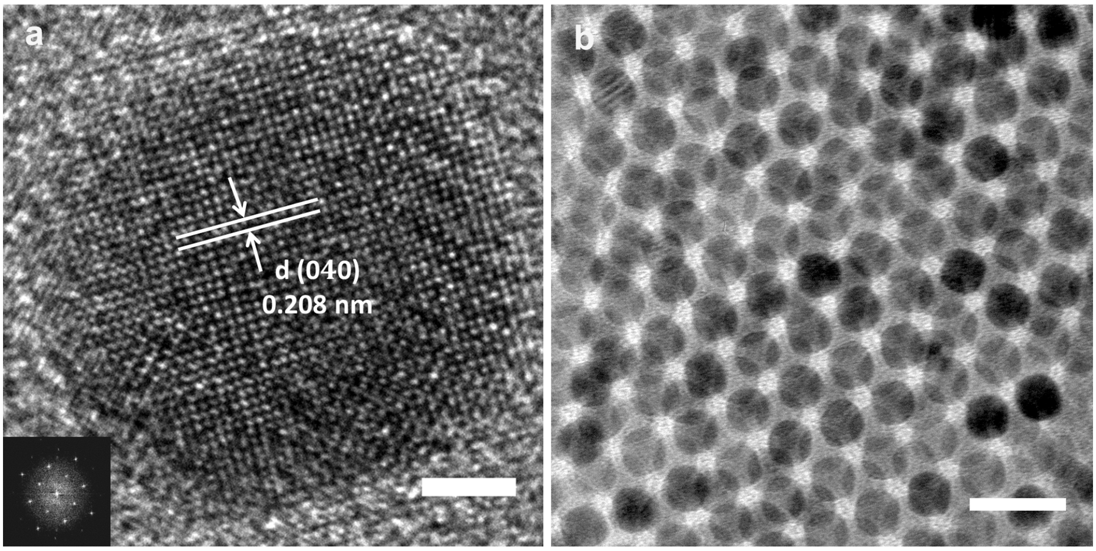

Supplementary Figure 2: EM micrographs of rounded $Fe_xO/CoFe_2O_4$ nanocubes. a) HRTEM image of a rounded $Fe_xO/CoFe_2O_4$ nanocube and b) self-assembled bilayer of rounded $Fe_xO/CoFe_2O_4$ nanocubes. FFT of the entire image is provided as an inset in bottom left corner of each image. Scale bars, a) 2 nm and b) 20 nm.

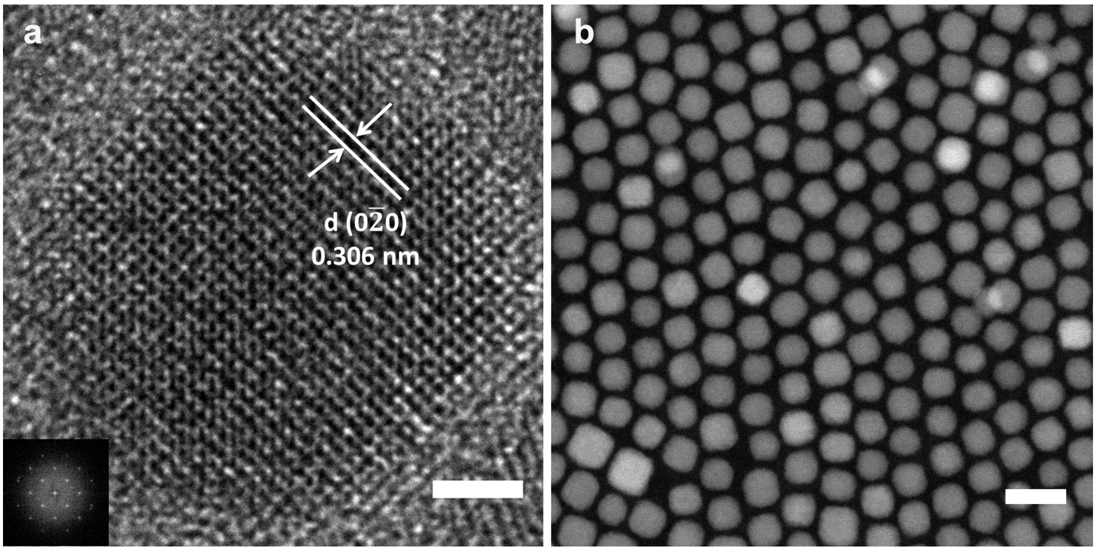

Supplementary Figure 3: EM micrographs of rounded PbSe nanocubes. a) HRTEM image of a rounded PbSe nanocube. Inset, FFT of HRTEM image is provided in the bottom left corner and b) HAADF-STEM image of self-assembled single layer of rounded PbSe nanocubes. Scale bars, a) 2 nm and b) 20 nm.

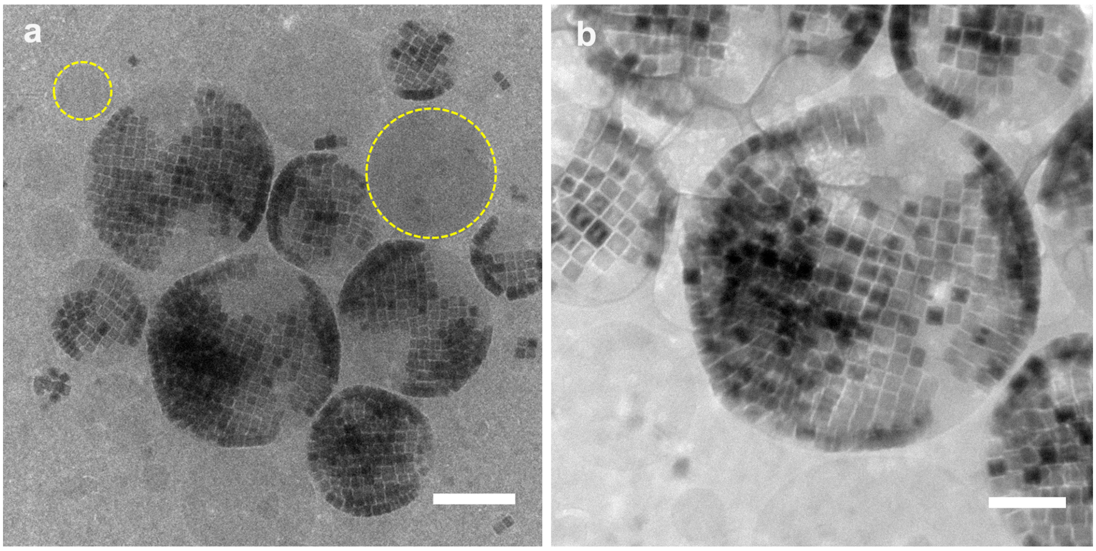

Supplementary Figure 4: Cryo-TEM images of freshly prepared cyclohexane-in-water emulsion droplets containing sharp Fe_3O_4 nanocubes. Empty cyclohexane-in-water emulsion droplets are indicated by the yellow dashed circles. Scale bars, a) 200 nm and b) 100 nm.

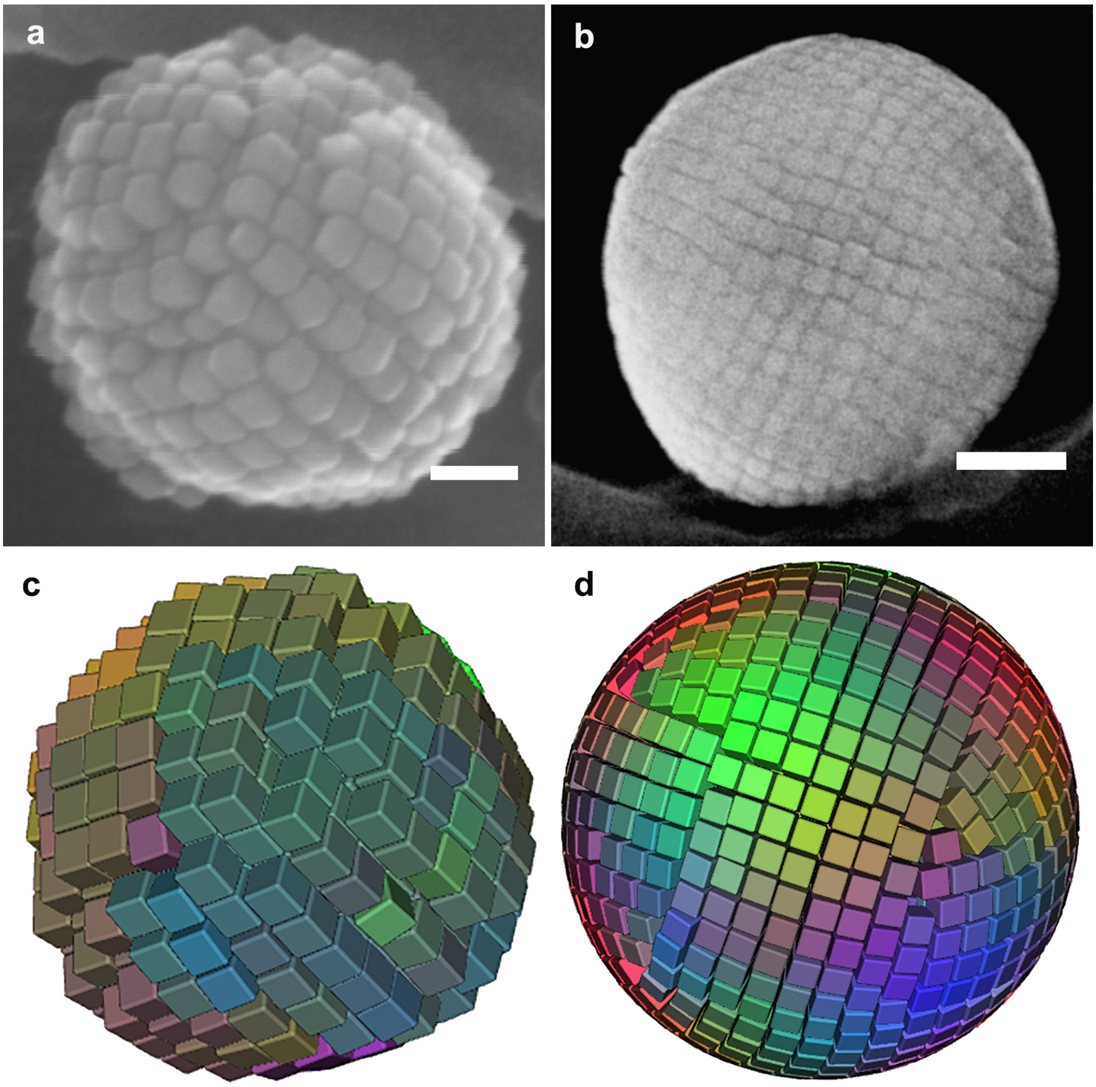

Supplementary Figure 5: SPs consisting of sharp Fe_3O_4 nanocubes. a) SEM image of a detached core with twisted SC lattice, b) cryo-SEM image of a sharp Fe_3O_4 nanocubes SP, showing its surface termination (note that the relatively low contrast is due to vitrification of the aqueous SP dispersion in cryo environment), c) core and d) surface terminations as obtained from the computer simulations with 2,000 particles in spherical confinement ($\alpha = 0.8$). For an interactive 3D view of d, see Supplementary Data 1. Scale bars, a) 50 nm and b) 100 nm.

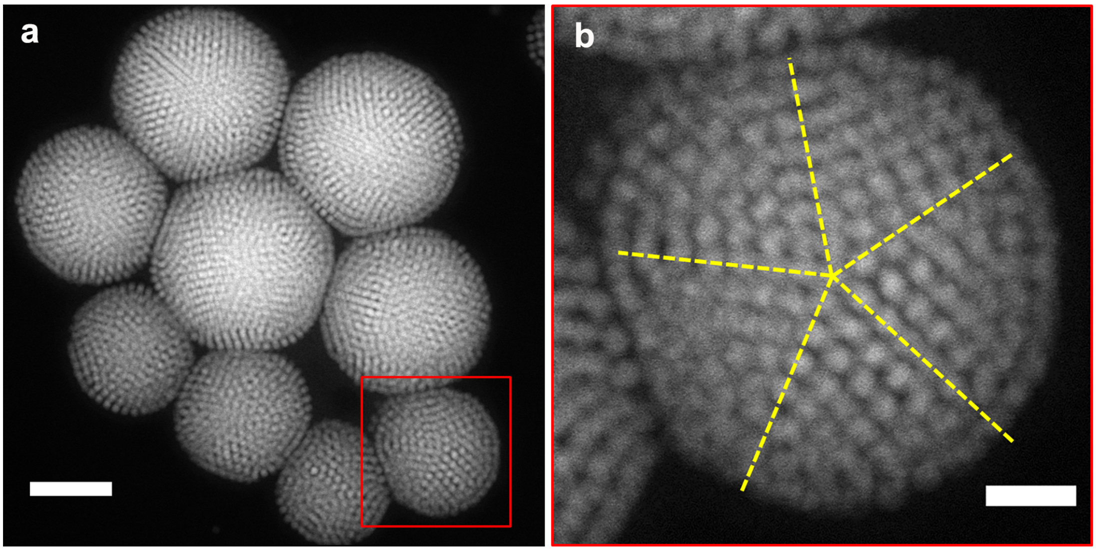

Supplementary Figure 6: HAADF-STEM images of SPs consisting of rounded $Fe_xO/CoFe_2O_4$ nanocubes. a) Overview of SPs and b) a single SP assembled from rounded $Fe_xO/CoFe_2O_4$ cubes with five-fold symmetry, as illustrated by the yellow dashed lines. Scale bars, a) 100 nm and b) 50 nm.

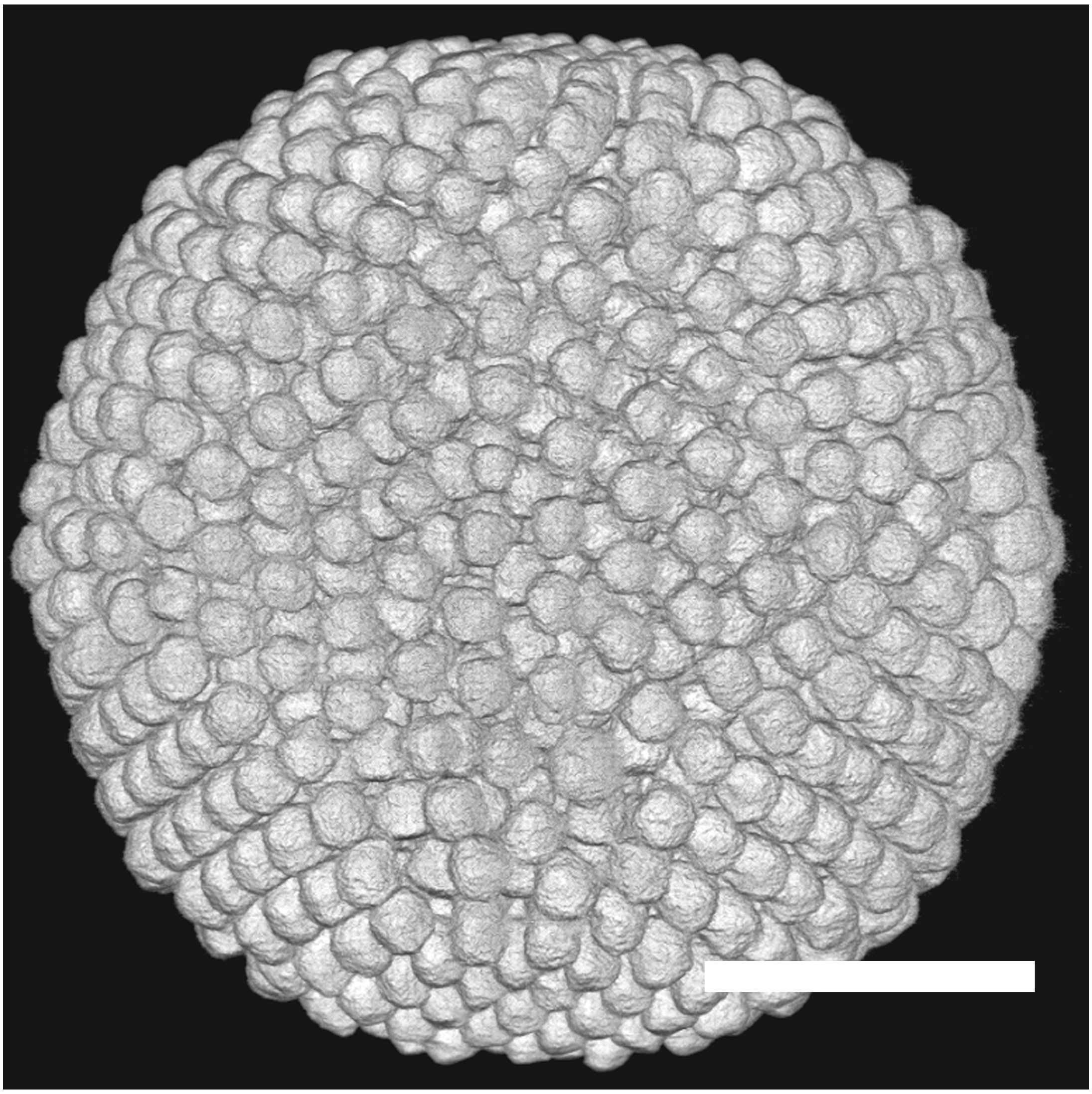

Supplementary Figure 7: 3D representation of the tomographic reconstruction of a SP consisting of rounded $Fe_xO/CoFe_2O_4$ nanocubes. Scale bar, 50 nm.

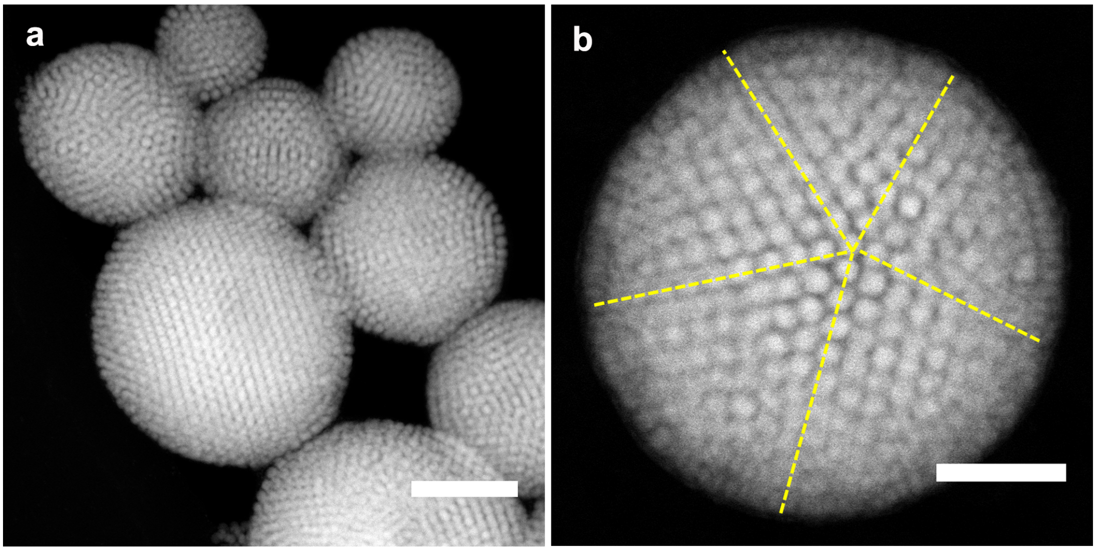

Supplementary Figure 8: EM micrographs of SPs consisting of rounded PbSe nanocubes. a) Overview of SPs and b) HAADF-STEM image of a single SP assembled from rounded PbSe nanocubes with five-fold symmetry, as illustrated by the yellow dashed lines. Scale bars, a) 100 nm and b) 50 nm.

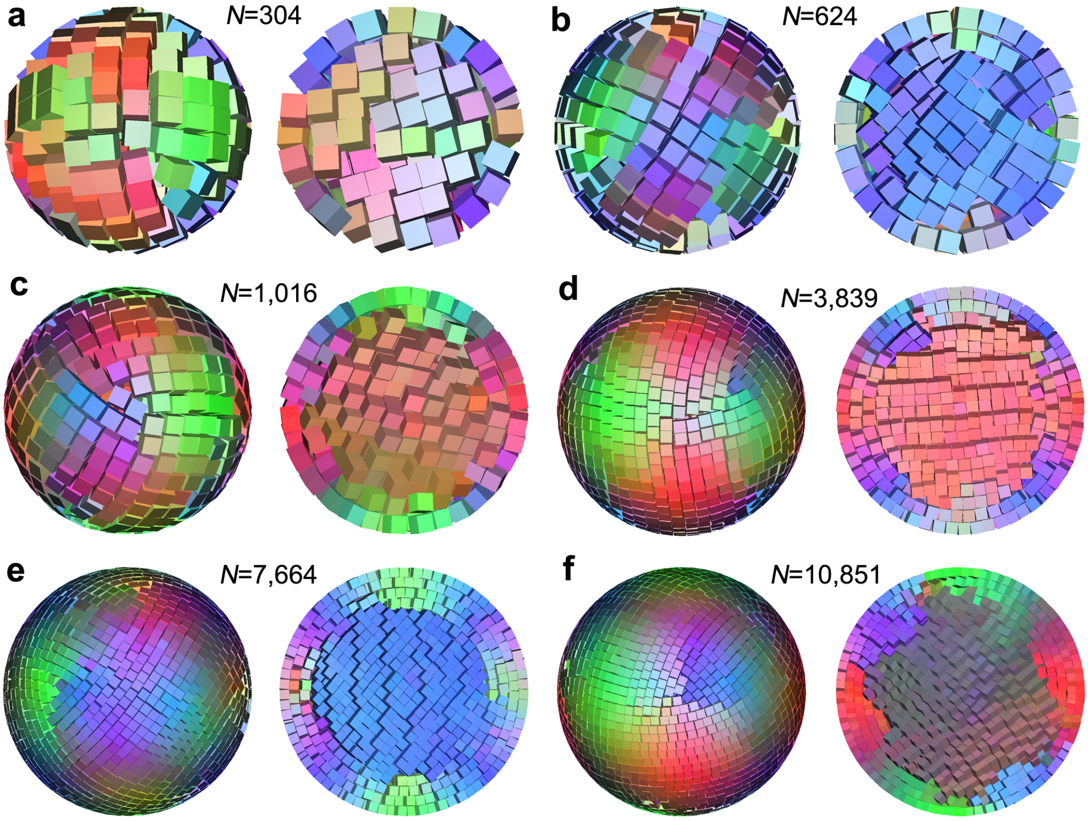

Supplementary Figure 9: Clusters from computer simulations (α = 1.0) with different sizes. Final configurations of compression for different system sizes, N. a) $N = 304$, b) $N = 624$, c) $N = 1,016$, d) $N = 3,839$, e) $N = 7,664$ and f) $N = 10,851$. The left and right column of each pair, shows a surface and cut-through view, respectively. Particles are colored according to their orientations.

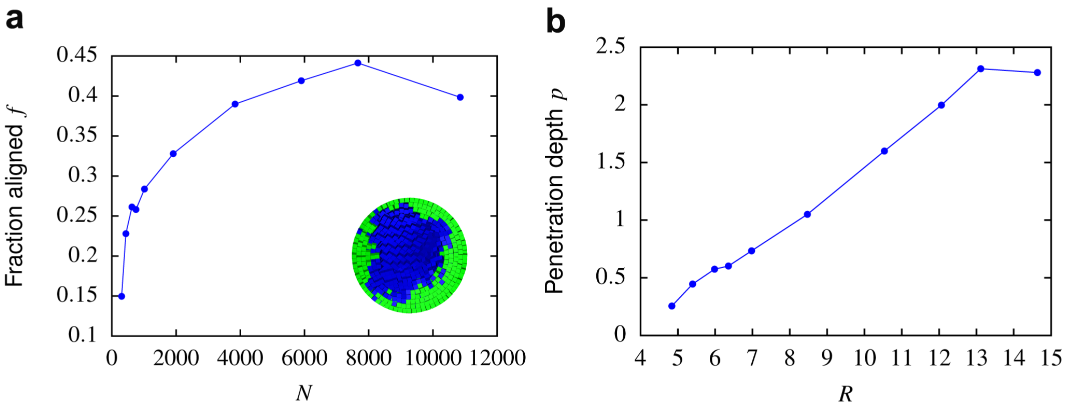

Supplementary Figure 10: Size dependence of shell thickness ($\alpha = 1.0$). a) The fraction of particles that ended up being aligned with the confining sphere as a function of the total number of particles. We used a threshold value for aligned of 0.07 radians between a normal of the cube and the normal of the closest point on the confining sphere. b) The penetration depth p as a function of the radius R of the SP. Inset, illustration of aligned particles (green) for $N = 7,664$.

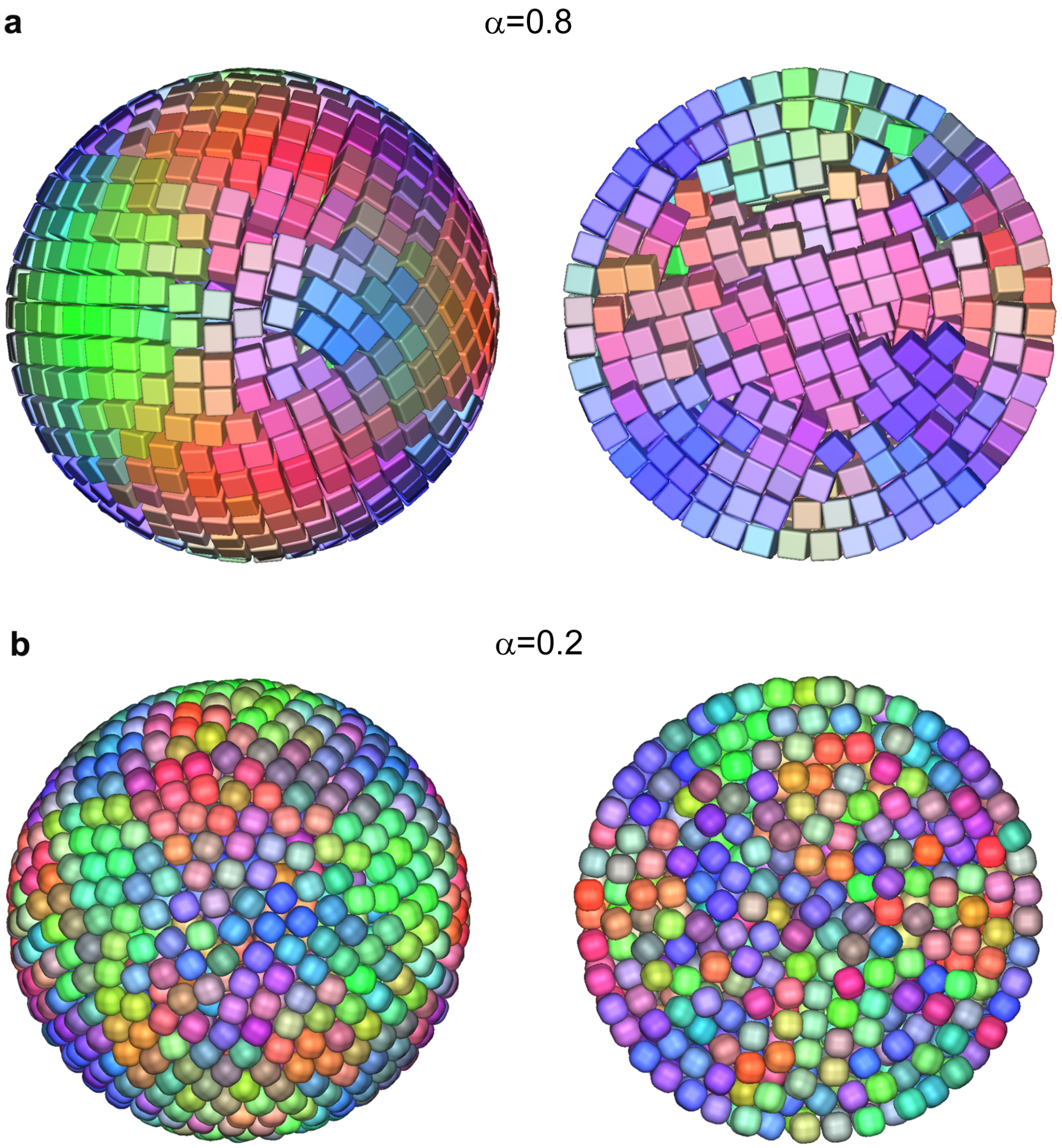

Supplementary Figure 11: Clusters from computer simulations with fast compression. Final configurations of compression with 2,000 particles with two different shape parameter α, in spherical confinement with high compression rate: a) $\alpha = 0.8$ and b) $\alpha = 0.2$. The left and right column of each pair, shows a surface and cut-through view, respectively. Particles are colored according to their orientations.

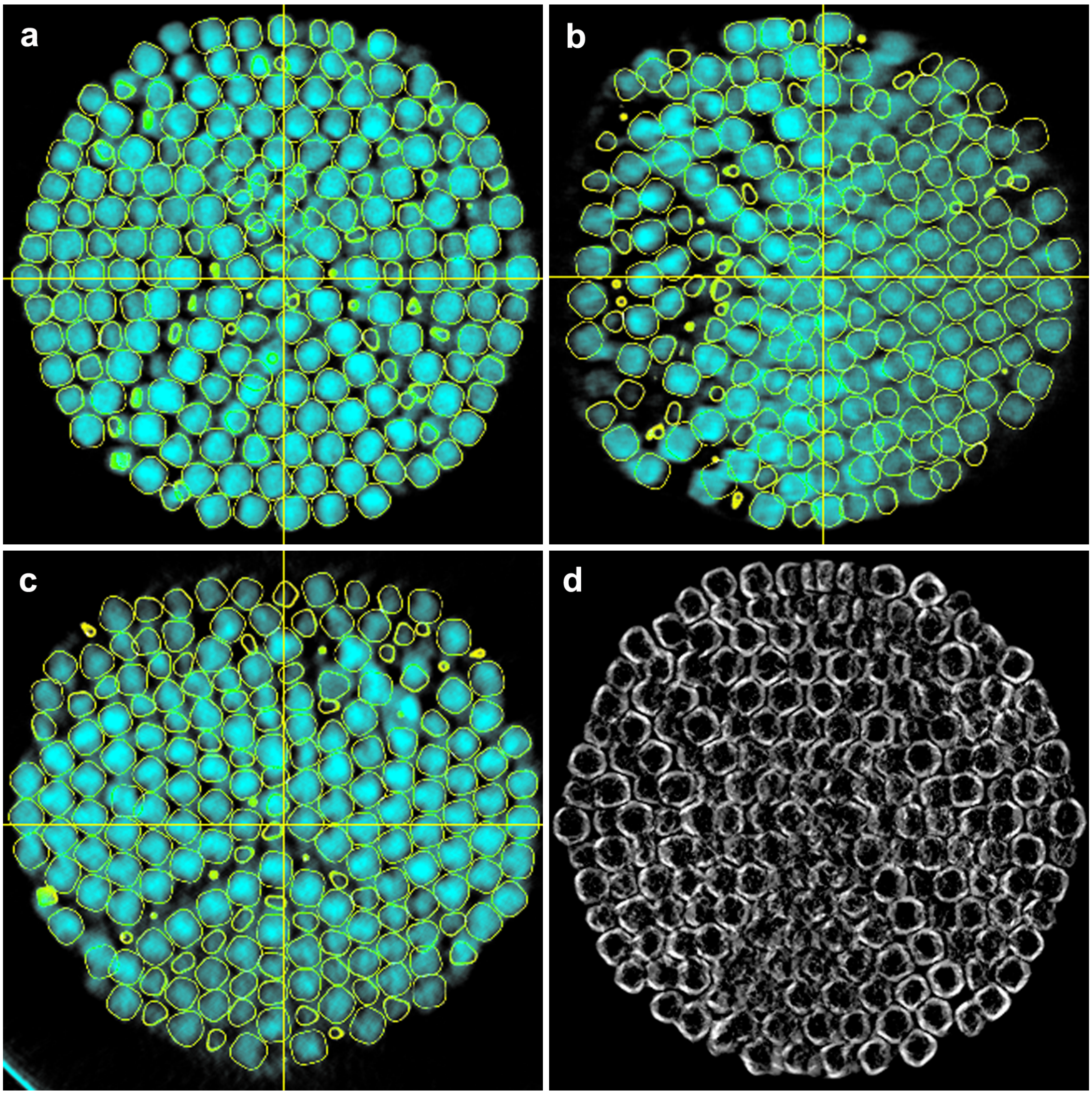

Supplementary Figure 12: Particle tracking. The reconstructed data is shown in cyan and the outlines of the particles found are shown in yellow. Three different slices through the same 3D data stack. The yellow cross shows the position of the other slices. a) XY image, b) YZ image, c) XZ image and d) XY gradient or edge detect image.

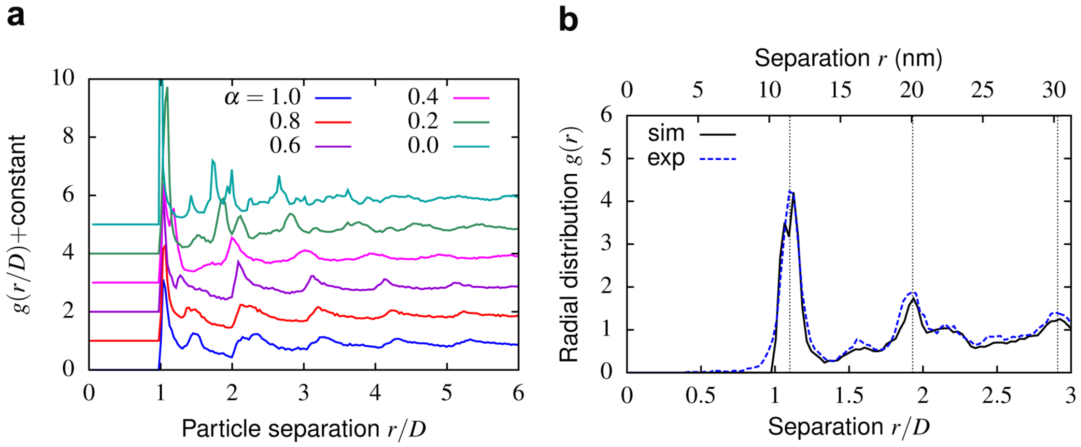

Supplementary Figure 13: The radial distribution function (offset by 1 for clarity). a) For $\alpha = 1.0, 0.8, 0.6, 0.4, 0.2$ and 0.0 as labeled. b) The experimental data of rounded cubes and the simulated $g(r)$ for $\alpha = 0.3$.

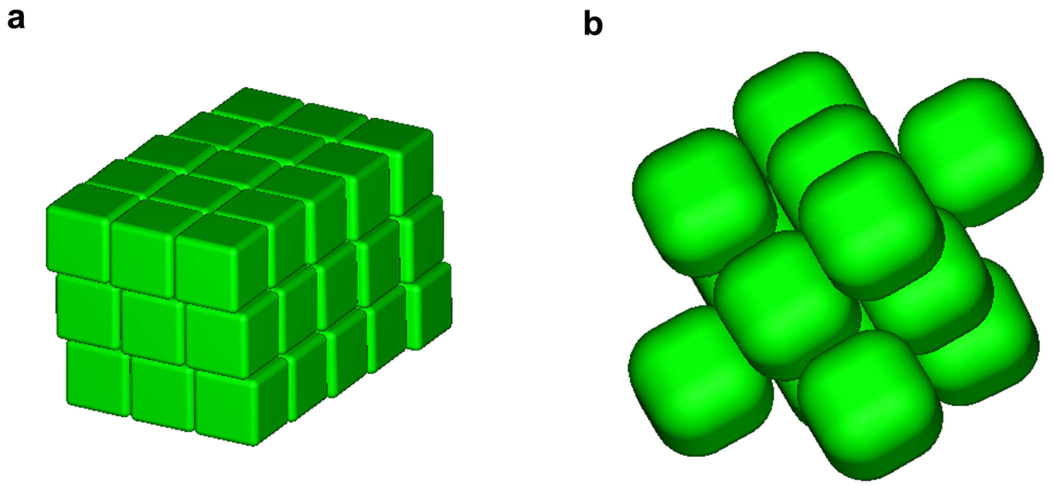

Supplementary Figure 14: Bulk close packed crystal structures of sharp and rounded cubes. a) The C1 lattice as obtained from bulk simulations with $\alpha = 0.8$ and b) the C0 lattice as obtained from bulk simulations with $\alpha = 0.3$.

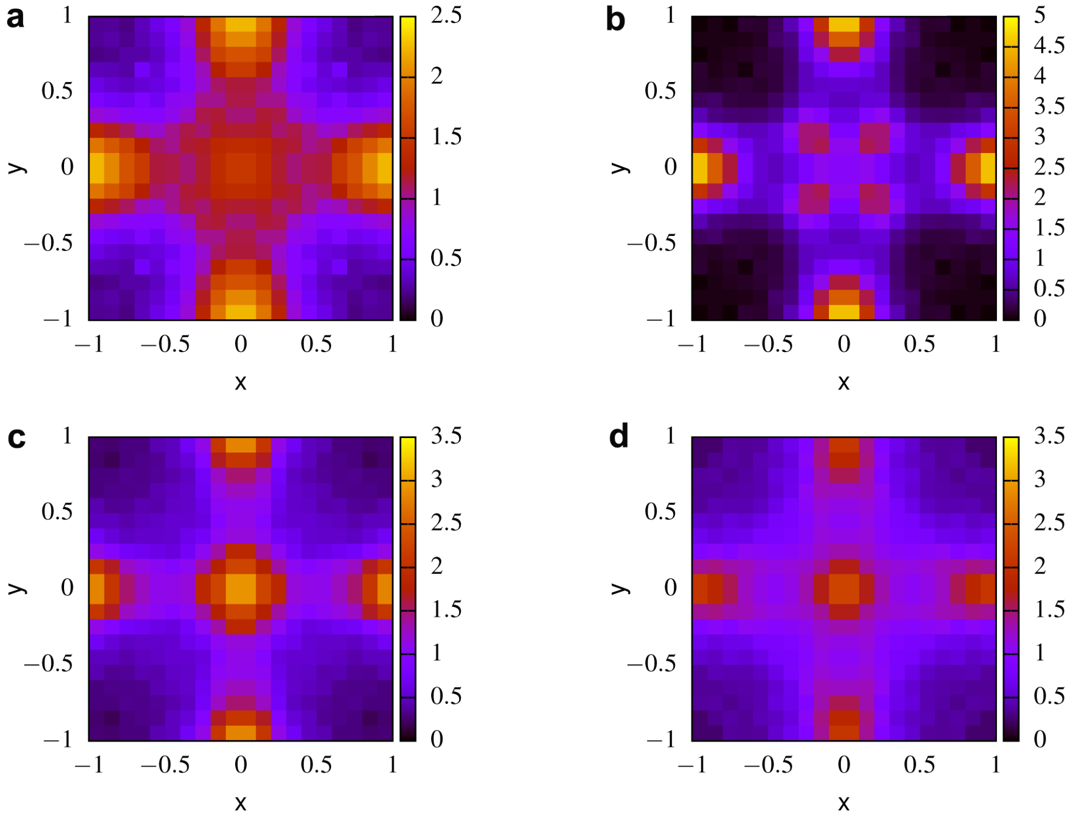

Supplementary Figure 15: Neighbor distributions for experiments and simulations. a) From bulk simulations with $\alpha = 0.3$ in the plastic crystal phase. b) From bulk simulations with $\alpha = 0.3$ in the C0 phase. c) From computer simulations in spherical confinement with $\alpha = 0.3$. d) From the experiments with rounded cubes. The bright (yellow) spot in the middle corresponds to neighbors positioned at the center of a face, the four bright spots in the middle of the edges correspond to neighbors oriented along one of the ribs of the rounded cube. Note that all the units are dimensionless as same as those of the radial distribution function shown in Supplementary Figure 13.

Supplementary References

1. Park, J. *et al.* Ultra-large-scale syntheses of monodisperse nanocrystals. *Nat. Mater.* **3,** 891–895 (2004).
2. Bodnarchuk, M. I. *et al.* Exchange-coupled bimagnetic wüstite/metal ferrite core/shell nanocrystals: size, shape, and compositional Control. *Small* **5,** 2247–2252 (2009).
3. Steckel, J. S., Yen, B. K. H., Oertel, D. C. & Bawendi, M. G. On the mechanism of lead chalcogenide nanocrystal formation. *J. Am. Chem. Soc.* **128,** 13032–13033 (2006).
4. Choi, J. J. *et al.* Controlling nanocrystal superlattice symmetry and shape-anisotropic interactions through variable ligand surface coverage. *J. Am. Chem. Soc.* **133,** 3131–3138 (2011).
5. Mason, T. G. & Bibette, J. Shear rupturing of droplets in complex fluids. *Langmuir* **13,** 4600–4613 (1997).
6. Gilbert, E. G., Johnson, D. W. & Keerthi, S. S. A fast procedure for computing the distance between complex objects in three-dimensional space. *IEEE J. Robot. Autom.* **4,** 193–203 (1988).
