# Supplementary material for: Interplay between spherical confinement and particle shape on the self-assembly of rounded cubes
Source: Nat Commun. 2018 Jun 8;9:2228. doi: 10.1038/s41467-018-04644-4 (PMC5994693; doi:10.1038/s41467-018-04644-4)
Supplement: Supplementary file 3 — Description of Additional Supplementary Information [file 41467_2018_4644_MOESM3_ESM.pdf]

**Description of Additional Supplementary Files**

File Name: Supplementary Data 1
Description: Simulation snapshot of 2,000 sharp cubes (α=0.8) confined in a sphere. The color indicates the orientation. The slider at the bottom can be used to visualize the inside.

File Name: Supplementary Data 2
Description: Interactive visualization of a SP consisting of sharp nanocubes (α=0.8). The red cubes highlight the topological defects on the surface of the SP

File Name: Supplementary Data 3
Description: Positions and orientations obtained from an experiment with rounded nanocubes (α=0.3) confined in a sphere. The color indicates the orientation. The slider at the bottom can be used to visualize the inside.

File Name: Supplementary Data 4
Description: Simulation snapshot of 2,000 rounded cubes (α=0.3) confined in a sphere. The color indicates the orientation. The slider at the bottom can be used to visualize the inside.

File Name: Supplementary Data 5
Description: Interactive visualization of the SP consisting of rounded nanocubes (α=0.3) obtained from an experiment. The color indicates the local order, dark blue is FCC, green is HCP and cyan is random or fluid like. The slider at the bottom can be used to visualize the inside.

File Name: Supplementary Data 6
Description: Simulation snapshot of 2,000 perfect cubes (α=1.0) confined in a sphere. The color indicates the orientation. The slider at the bottom can be used to visualize the inside.

File Name: Supplementary Data 7
Description: Simulation snapshot of 2,000 sharp cubes (α=0.6) confined in a sphere. The color indicates the orientation. The slider at the bottom can be used to visualize the inside.

File Name: Supplementary Data 8
Description: Simulation snapshot of 2,000 rounded cubes (α=0.4) confined in a sphere. The color indicates the orientation. The slider at the bottom can be used to visualize the inside.

File Name: Supplementary Data 9
Description: Simulation snapshot of 2,000 rounded cubes (α=0.2) confined in a sphere. The color indicates the orientation. The slider at the bottom can be used to visualize the inside.

File Name: Supplementary Movie 1
Description: A tomography tilt series and reconstruction of a supraparticle composed of rounded $Fe_xO/CoFe_2O_4$ nanocubes.

File Name: Supplementary Movie 2
Description: 3D representation of the reconstructed supraparticle.
