# Supplementary material for: Interplay between spherical confinement and particle shape on the self-assembly of rounded cubes
Source: Nat Commun. 2018 Jun 8;9:2228. doi: 10.1038/s41467-018-04644-4 (PMC5994693; doi:10.1038/s41467-018-04644-4)
Supplement: Supplementary file 5 — Supplementary Data 2 [file 41467_2018_4644_MOESM5_ESM.html]

Supplementary figures


## Supplementary Data 2

Interactive visualization of a SP consisting of sharp nanocubes (α=0.8).
The red cubes highlight the topological defects on the surface of the SP.

Made using  Visual colloids.
