# Supplementary material for: Interplay between spherical confinement and particle shape on the self-assembly of rounded cubes
Source: Nat Commun. 2018 Jun 8;9:2228. doi: 10.1038/s41467-018-04644-4 (PMC5994693; doi:10.1038/s41467-018-04644-4)
Supplement: Supplementary file 8 — Supplementary Data 5 [file 41467_2018_4644_MOESM8_ESM.html]

Supplementary figures


## Supplementary Data 5

Interactive visualization of the SP consisting of rounded nanocubes (α=0.3) obtained from an experiment.
The color indicates the local order, dark blue is FCC, green is HCP and cyan is random or fluid like.
The slider at the bottom can be used to visualize the inside.

Made using  Visual colloids.
