# Supplementary material for: Interplay between spherical confinement and particle shape on the self-assembly of rounded cubes
Source: Nat Commun. 2018 Jun 8;9:2228. doi: 10.1038/s41467-018-04644-4 (PMC5994693; doi:10.1038/s41467-018-04644-4)
Supplement: Supplementary file 10 — Supplementary Data 7 [file 41467_2018_4644_MOESM10_ESM.html]

Supplementary figures


## Supplementary Data 7

Simulation snapshot of 2,000 sharp cubes (α=0.6) confined in a sphere. The color indicates the orientation. The slider at the bottom can be used to visualize the inside.

Made using  Visual colloids
